# The Potential Role of Glucagon-Like Peptide-1 (GLP-1) Agonists for Polycystic Ovary Syndrome

**DOI:** 10.7759/cureus.77998

**Published:** 2025-01-26

**Authors:** Shaima Rahim, Joseph Pergolizzi

**Affiliations:** 1 Research, NEMA Research, Inc., Naples, USA; 2 Pharmacy, Temple University, Philadelphia, USA; 3 Pain Management, NEMA Research, Inc., Naples, USA

**Keywords:** fertility, glp-1 receptor agonists, glucagon-like peptide-1 receptor agonists, insulin resistance, obesity, polycystic ovary syndrome, safety concerns, weight loss

## Abstract

Polycystic ovary syndrome (PCOS) poses a multifaceted challenge, affecting women through genetic susceptibility, obesity, and insulin resistance. This narrative review explores the potential therapeutic role of glucagon-like peptide-1 receptor agonists (GLP-1 RAs) in PCOS treatment, with a focus on weight loss and associated metabolic changes. The off-label use of GLP-1 RAs in this population helps to treat comorbid obesity. By thoroughly examining PCOS diagnostic criteria, current treatments, and clinical trial outcomes involving GLP-1 RAs, this research reveals encouraging results. However, concerns about the long-term safety of GLP-1 RAs, including serious adverse events, warrant further investigation. While GLP-1 RAs hold promise for treating obesity in PCOS, safety issues may limit their utility.

## Introduction and background

Polycystic ovary syndrome (PCOS) is the most widely recognized endocrine problem that affects up to 18% of women of reproductive age, affecting up to 5 million women in the United States [1,2]. It is a polygenic, polyfactorial, systemic, inflammatory, dysregulated steroid state, and autoimmune disease [3] with 70% heritability [2]. Many women with PCOS (38%-88%) are either overweight or obese and have insulin resistance (50%-90%) [4]. Various reproduction disorders such as hyperandrogenemia, imbalanced gonadotropin secretion, ovulatory dysfunction, polycystic ovarian morphology, infertility, miscarriages, premature birth, and gestational diabetes are also seen in patients [5]. Women with PCOS have decreased growth hormone (GH), which can result in increased visceral adiposity (VAT) and impaired vascular function [6]. Adiposity strongly influences PCOS phenotypes and worsens the management of symptoms and fertility outcomes [4].

**Figure 1:**
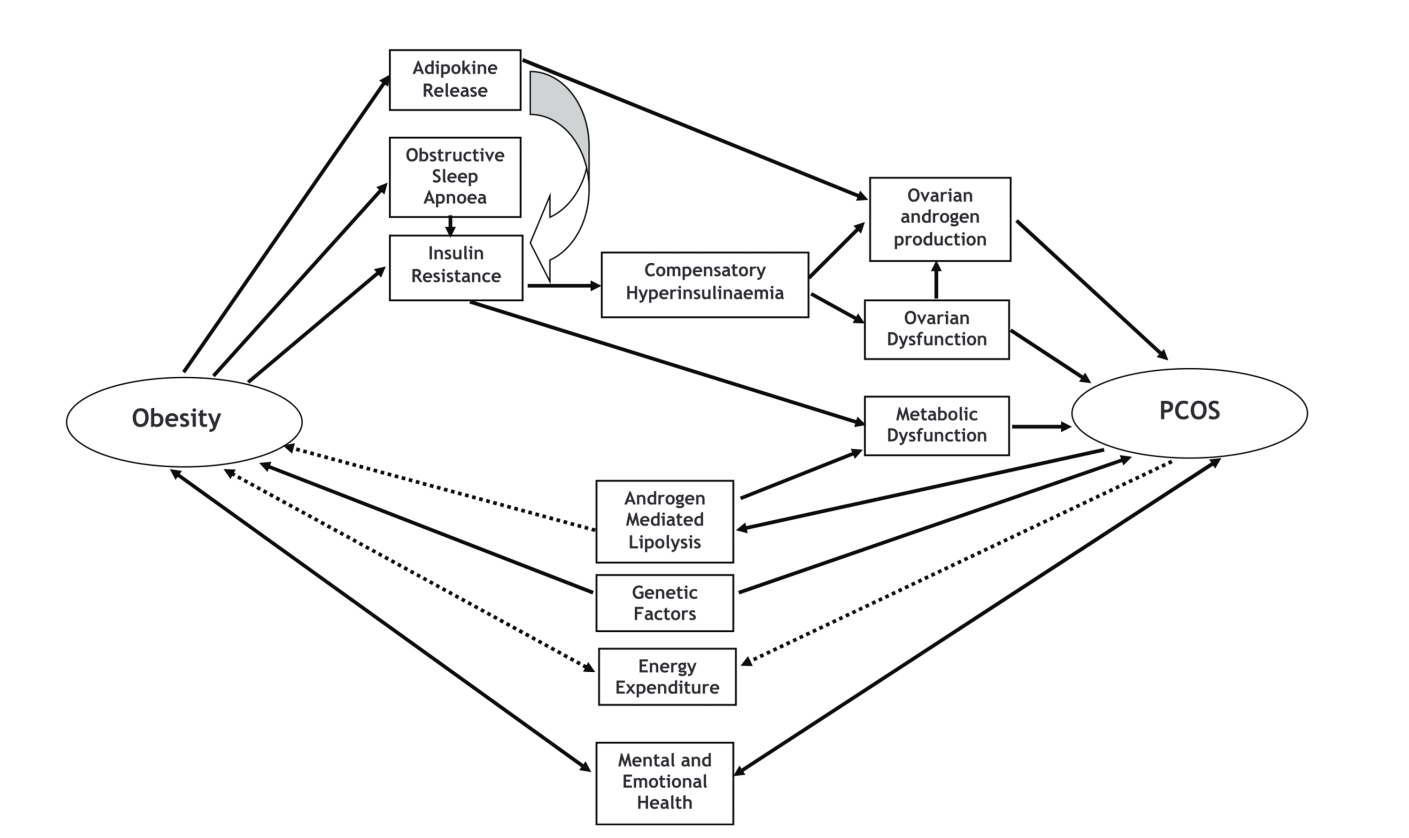
The interconnected and complex relationship between obesity and polycystic ovary syndrome. Chart made available courtesy of SAGE Publications, Barber TM, Hanson, P, Weicker MO, Franks S. Obesity and Polycystic Ovary Syndrome: Implications for Pathogenesis and Novel Management Strategies [4]. PCOS: polycystic ovary syndrome

There is an interconnected relationship between obesity and PCOS as demonstrated by Figure 1. The complexity of PCOS and the array of comorbidities associated with it is seen as interlinked with those of obesity. Therefore, no one method of treatment can work for every patient but a multifaceted approach catered to each individual depending on their symptoms, would be deemed beneficial.

There is no one treatment for this disorder, but often a symptomatic approach is used to make prescribing choices [5]. Controlling weight gain and losing body fat is an innovative approach. Since PCOS and obesity-induced insulin resistance are comorbid, trials of diabetes drugs in PCOS patients are ongoing [5]. The prevalence of type 2 diabetes mellitus (T2DM) in women with PCOS ranges from 1.5 to 12.4 %. However, glucagon-like peptide-1 receptor agonists (GLP-1 RA), typically used in the treatment of T2D, have been considered as an off-label PCOS therapy for women who do not have T2DM [7]. These drugs simultaneously target associated conditions and phenotypic presentations of PCOS as these agents improve insulin sensitivity, reduce cardiovascular disease risk, result in weight loss, and improve nonalcoholic fatty liver disease [7]. Many of the listed indications for GLP-1 RAs seem appropriate for the symptoms associated with PCOS.

Methods

A search on PCOS was first done separately on PubMed to understand the diagnostics, epidemiology, and morbidities associated with the syndrome in October of 2023. Next, the GLP-1 drugs were researched on the same search engine. Finally, an advanced search consisting of “PCOS” and “GLP- 1” drugs was made on PubMed and the data was limited to randomized clinical trials. This search yielded 24 results. Human studies, articles in English, and articles that tested GLP-1 drugs were included. Articles and studies on pediatric patients were excluded. All 24 results were analyzed to search for relevant information. Among them, the most relevant results were summarized along with the significance and correlation between GLP-1 analogs on PCOS and the adverse effects. Additionally, a separate search on the “serious adverse events” of “GLP-1” drugs was performed for information on general adverse events outside of PCOS-specific trials.

## Review

Results

Polycystic Ovary Syndrome (PCOS) Diagnostic Criteria

**Figure 2:**
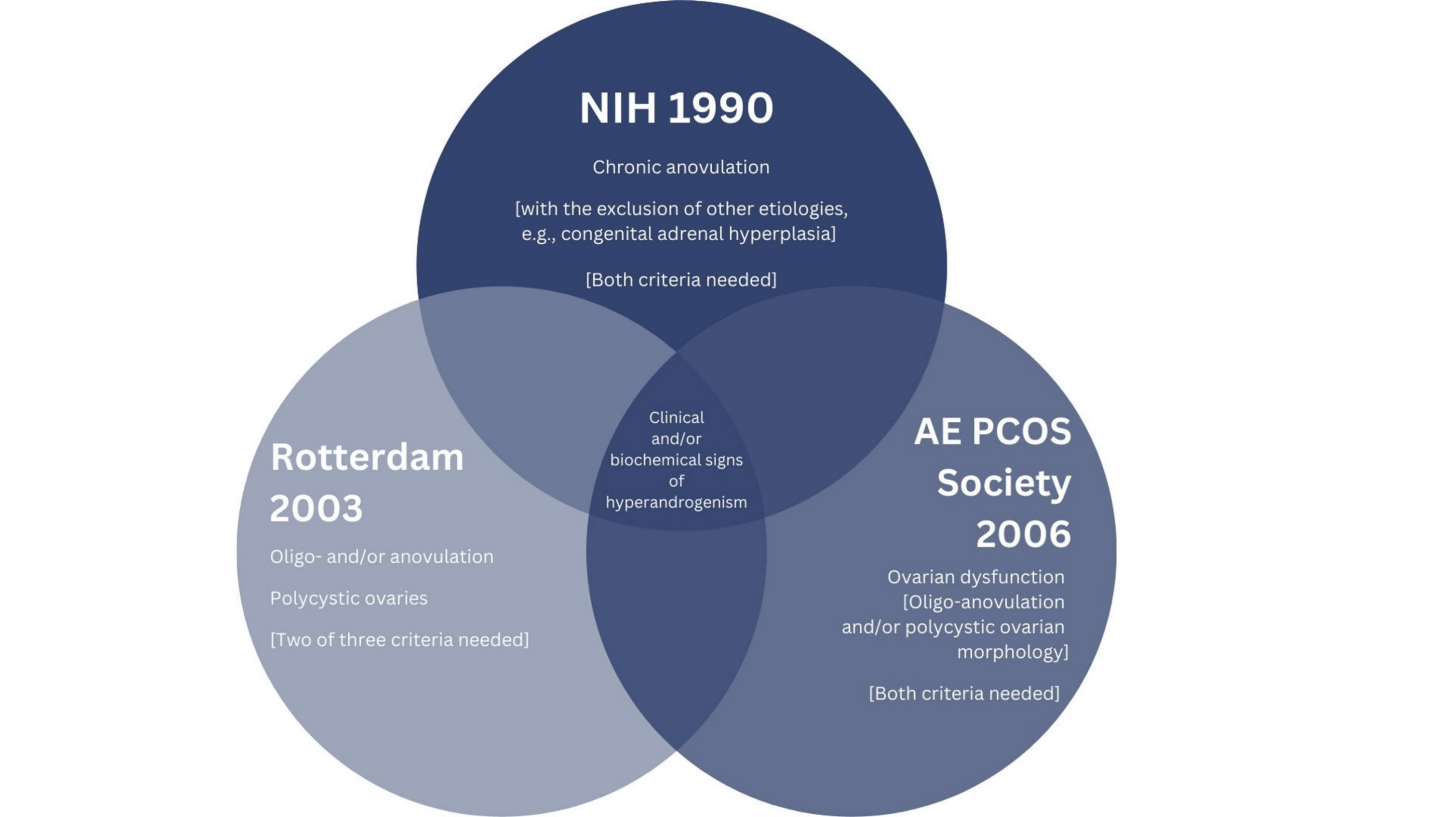
Diagnostic criteria for polycystic ovary syndrome. There are currently three known diagnostic criteria for polycystic ovary syndrome. The National Institute of Health (NIH) 1990, Rotterdam 2003, and Androgen Excess & Polycystic Ovary Syndrome Society (AE-PCOS) 2006. These criteria have recently been reviewed [8]. The image was created by the author (Rahim S) of this article.

All the trials analyzed in this review use one or more panels from the diagnostic criteria mentioned below in Figure 2 to diagnose their patients with PCOS. All three diagnostic criteria focus on three main panels: Excessive androgen (hyperandrogenism), ovulatory dysfunction (oligo-anovulation), and polycystic ovarian morphology (polycystic ovaries). Most controlled trials use one of these three to define PCOS. Note that with the NIH 1990 criteria, chronic anovulation is not a criterion if it is caused by other etiologies like congenital adrenal hyperplasia [8].

Treatments

Lifestyle changes, such as diet and physical activity, as the first line of treatment for obesity in this population, have not been especially effective [9]. However, the application of pharmacotherapy in combination with lifestyle modifications enhanced weight loss efficiency [9]. GLP-1 RAs may be useful for weight loss and eating disorders through improved glucose homeostasis and reduced food intake by a direct hypothalamic effect of the hormone [10]. Women with PCOS are at elevated risk for eating disorders [11].

GLP-1 is an incretin hormone that is secreted from the intestine postprandial via the incretin effect. This incretin hormone is inactivated by dipeptidyl peptidase-4 (DPP-4). This incretin effect and its regulation must be maintained for proper glucose homeostasis, but it is muted or absent in people with T2D [12,13]. GLP-1 RA amplifies the endogenous secretion of insulin leading to improved glucose homeostasis. This improvement can further inhibit appetite and result in changed eating behaviors, thereby promoting weight loss in patients at a greater rate than any other glucose-lowering agents [10]. There are many approved GLP-1 RAs on the market that are either short-acting or long-acting. Most of the studies performed used the long-acting liraglutide, short-acting exenatide, and short-acting recombinant human GLP-1 known as beinaglutide. These drugs and/or metformin have been used in PCOS trials.

Several studies examined various weight loss and metabolic outcomes in obese women diagnosed with PCOS. Different therapeutic regimens comprising GLP-1 RAs were explored. Elkind-Hirsch et al. [9] found that liraglutide daily resulted in a 5.7% weight loss, with improvements in the free androgen index and improved menstrual cycles compared to placebo. Sever et al. [10] found that the combination of metformin and liraglutide resulted in the greatest reductions in BMI and waist circumference compared to either drug alone. Liu et al. [14] reported that exenatide led to greater fat reduction, improved insulin resistance, increased menstrual frequency ratio, and increased rate of natural pregnancy compared to metformin. Salamun et al. [15] showed that combining metformin with liraglutide led to weight loss and higher pregnancy success rates compared to metformin alone. Wen et al. [16] found that the combination of metformin with semaglutide resulted in greater weight loss and reductions in BMI and testosterone levels compared to metformin alone. In contrast, Elkind-Hirsch, et al. [17] showed that exenatide weekly and dapagliflozin, either alone or combined, achieved more significant weight loss than other treatments. However, the combination of exenatide and dapagliflozin produced the greatest effect. Jensterle et al. [18] noted that liraglutide alone was more effective than a combination of metformin and liraglutide for weight loss, but the combination therapy showed improved androgen profiles and tolerability. Ma et al. [19] found that the addition of exenatide to metformin significantly contributed to weight loss and reduced waist circumference. Additionally, Jensterle et al. [20] reported that liraglutide treatment led to a decrease in uncontrolled eating (UE) scores from 36.8 ± 24.5 to 19.6 ± 18.4 (p < 0.001) and a decrease in emotional eating (EE) score from 49.9 ± 33.3 to 28.5 ± 26.9 (p < 0.001) in obese women with PCOS [20].

Across all the trials that were analyzed, the adverse events that occurred were mild to moderate gastrointestinal side effects. The most common adverse effects were nausea, vomiting, and diarrhea during the first 4-8 weeks of treatment. Intermittent itching was reported in one trial that used liraglutide [16]. One woman reported gall bladder-related concerns a week after the end of the therapy [18]. There were no unexpected adverse events that occurred.

Discussion

Treating PCOS with GLP-1 RA is an off-label indication. The strength of this study is that we are exploring an off-label use of a very popular drug class. The FDA initially approved GLP-1 analogs for treating T2DM. Both semaglutide and long-acting liraglutide were approved by the FDA only recently for obesity [12]. This resulted in increased demand for these drugs. The USD 12 billion market in 2023 is expected to increase. The COVID-19 pandemic positively impacted the GLP-1 Agonists Market [21].

Results from various trials show the benefits of GLP-1 RA drugs when it comes to weight loss in PCOS patients as well. However, other metabolic changes were also observed within the short period like reduction in BMI, waist circumference reduction, improved menstrual frequency, and pregnancy rates among many others. Longer exposure to these drugs could potentially give rise to more metabolic improvements. Additionally, combining GLP-1 RA drugs with metformin brought on more improvements than using metformin for PCOS alone. Treatment of PCOS as well as T2DM with metformin is a popular primary choice of treatment before considering GLP-1 analogs [12,22]. However, the costs for these drugs may make them unavailable for those without health insurance or whose insurance will not cover the drugs for this off-label use.

The long-term safety of GLP-1 RAs for weight management remains unknown. While promising in the short term, animal studies and direct patient reports raise concerns. Thus, more study is needed to better understand the long-term safety implications of these agents. There may be a higher incidence of serious infections, such as COVID-19 pneumonia and common upper respiratory/urinary tract infections with GLP-1 RAs [23]. Nasopharyngitis, influenza, cystitis, and viral infection commonly have also been reported with these drugs [24]. While headaches have been frequently reported in trials, the broader spectrum of neurological effects requires further investigation. These findings highlight the need for more comprehensive long-term studies to fully understand the safety profile of these drugs [24,25]. Concerns regarding potential thyroid cancer risk with GLP-1 mimetic therapies, particularly exenatide, arose following reports of increased thyroid tumors in rodents treated with liraglutide. There is little data on thyroid tumors from clinical studies, although it appears exenatide is associated with a greater risk than sitagliptin [25]. Incretin-mimetic therapies have been linked to a two-fold increased risk of pancreatitis. The specific drugs sitagliptin and exenatide have been associated with a six-fold increase in reported pancreatitis and a higher prevalence of pancreatic cancer compared to other GLP-1 RAs.

GLP-1 analogs are effective in the PCOS population for weight loss, but PCOS patients who wish to become pregnant are not indicated for this kind of treatment. However, these drugs are indicated for diabetes control which is prevalent in the PCOS population. They just may be inappropriate for PCOS patients with diabetes who seek to become pregnant. The package inserts for liraglutide and semaglutide advise against the use of these drugs in women who are trying to get pregnant [26,27]. They warn about major birth defects and miscarriages. This might be because there are no data available with GLP -1 RAs in pregnant women to inform us of the associated risks. Women with PCOS suffer from infertility and are put on these drugs off-label to get pregnant even though the manufacturer warns against it due to the teratogenic risk being unknown. Recent research has shown that the prevalence of major congenital malformations was lower among infants with periconceptional exposure to no antidiabetic medications (ADMs) (4.77%) or metformin only (5.32%) than among those exposed to insulin (7.83%), sulfonylureas (9.71%) and GLP-1 receptor agonists (8.23%). However, no direct link associated with major birth defects and GLP-1 RAs was established in the study [28]. The clinical trials above [15,14], present an increase in fertility rates due to GLP-1 RAs usage, but there are no long-term data to confirm if there were any major birth defects from those pregnancies. The animal studies in a recent review highlighted that GLP-1 agonists are excreted in breast milk and affect growth. They also affect fetal weight and skeletal ossification in animal studies but no human data is available [29]. There are limited human data on these medications, to guide decision-making regarding the timing of discontinuation of GLP-1 RA before conception [30]. Even finding data on the washout period could help prevent any fetal birth defects. Nonetheless, there are no updates from the manufacturing company.

According to the Centers for Disease Control and Prevention, the general fertility rate in the United States decreased by 3% from 2022, reaching a historic low [31]. This could lead to a potential increase in the prescribing of GLP-1s since clinical trials have pointed toward an improvement in fertility rates among women on GLP-1s with T2D, PCOS, or obesity [14,15]. There is robust evidence from clinical trials that show the multi-benefit nature of GLP-1 analogs but not enough information is out there about the long-term ramifications of these drugs. Just like the other reviews [28,30], this review ushers the importance of more long-term clinical research being conducted on these drugs due to 'the increase in periconceptional use of second-line noninsulin ADMs, particularly GLP-1 receptor agonists in the US, highlighting that there has been a shift in how T2D in reproductive-aged women is treated' [28]. Further studies must be done to understand the risks due to the increasing prescribing of these drugs.

This study has limitations. It is a literature review on new drugs for an off-label indication. There were few studies, and the studies were not large. All studies were short term and we do not have long-term data. In this paper, we compared a variety of products that may not all be equivalent. The costs of these drugs may make them unavailable for those without health insurance or whose insurance will not cover the drugs for this off-label use.

## Conclusions

PCOS is a prevalent condition that is associated with obesity. New antidiabetic drugs such as the GLP-1 RAs have been used in the PCOS population to help manage obesity, with the result that they confer other metabolic benefits as well. GLP-1 RAs appear in clinical studies to be more effective than metformin in weight loss in the PCOS population, but the combination of a GLP-1 RA plus metformin offers superior results. Long-term studies are needed to better evaluate potential adverse events, particularly with prolonged exposure. In short- and mid-term studies, most adverse events were mild to moderate. The role of these new GLP-1 RAs in PCOS patients appears promising but safety, availability, insurance coverage, and cost must be better evaluated.
